# Chromatin run-on sequencing analysis finds that ECM remodeling plays an important role in canine hemangiosarcoma pathogenesis

**DOI:** 10.1186/s12917-020-02395-3

**Published:** 2020-06-22

**Authors:** Chinatsu Mukai, Eunju Choi, Kelly L. Sams, Elena Zu Klampen, Lynne Anguish, Brooke A. Marks, Edward J. Rice, Zhong Wang, Lauren A. Choate, Shao-Pei Chou, Yukinari Kato, Andrew D. Miller, Charles G. Danko, Scott A. Coonrod

**Affiliations:** 1 Baker Institute for Animal Health, College of Veterinary Medicine, Cornell University, Ithaca, NY USA; 2 Department of Biomedical Sciences, Section of Anatomic Pathology, College of Veterinary Medicine, Cornell University, Ithaca, NY USA; 3 Department of Antibody Drug Development, Tohoku University Graduate School of Medicine, Sendai, Japan; 4 New Industry Creation Hatchery Center, Tohoku University, Sendai, Japan

**Keywords:** Hemangiosarcoma, ChRO-seq, ECM, Collagen, LAMA4, PDPN

## Abstract

**Background:**

Canine visceral hemangiosarcoma (HSA) is a highly aggressive cancer of endothelial origin that closely resembles visceral angiosarcoma in humans, both clinically and histopathologically. Currently there is an unmet need for new diagnostics and therapies for both forms of this disease. The goal of this study was to utilize Chromatin run-on sequencing (ChRO-seq) and immunohistochemistry (IHC) to identify gene and protein expression signatures that may be important drivers of HSA progression.

**Results:**

ChRO-seq was performed on tissue isolated from 17 HSA samples and 4 normal splenic samples. Computational analysis was then used to identify differentially expressed genes and these factors were subjected to gene ontology analysis. ChRO-seq analysis revealed over a thousand differentially expressed genes in HSA tissue compared with normal splenic tissue (FDR < 0.005). Interestingly, the majority of genes overexpressed in HSA tumor tissue were associated with extracellular matrix (ECM) remodeling. This observation correlated well with our histological analysis, which found that HSA tumors contain a rich and complex collagen network. Additionally, we characterized the protein expression patterns of two highly overexpressed molecules identified in ChRO-seq analysis, podoplanin (PDPN) and laminin alpha 4 (LAMA4). We found that the expression of these two ECM-associated factors appeared to be largely limited to transformed endothelial cells within the HSA lesions.

**Conclusion:**

Outcomes from this study suggest that ECM remodeling plays an important role in HSA progression. Additionally, our study identified two potential novel biomarkers of HSA, PDPN and LAMA4. Interestingly, given that function-blocking anti-PDPN antibodies have shown anti-tumor effects in mouse models of canine melanoma, our studies raise the possibility that these types of therapeutic strategies could potentially be developed for treating canine HSA.

## Background

Angiosarcomas (AS) are highly aggressive malignant tumors originating from endothelial cells. They account for approximately 2% of all soft tissue sarcomas in humans with the number of cases increasing significantly over the past 30 years [1–5]. For patients presenting with non-metastatic AS, the reported five-year survival rate is ~ 35%. These patients also have a 75% chance of local recurrence within 24 months and a 50% likelihood of metastases developing despite local treatment [4, 6]. When metastases are already present at the initial presentation, the five-year survival rate is poor, with a median survival time of just 3 months [4]. Although published research on this rare tumor is increasing, we still know very little about the pathogenesis of this disease.

Canine hemangiosarcoma (HSA) is histopathologically similar to angiosarcoma, with both forms of this disease following a similar clinical course [7]. Canine HSA is most commonly observed in the spleen, but also occurs in other organs such as the heart, liver, and dermis with the later form often being associated with ultraviolet radiation-associated oncogenesis. Prognosis for the visceral forms of HSA is poor, with most dogs dying from this disease within months of their diagnosis [8, 9]. For dogs diagnosed with splenic HSA, surgical removal of the spleen can increase the life expectancy up to 6 months, and when combined with chemotherapy, can prolong their life for up to 12 months [8, 9].

While all breeds are susceptible to hemangiosarcoma, German Shepherds, Golden Retrievers, and Labrador Retrievers, are particularly predisposed to developing this disease [10], with the estimated lifetime risk of Golden Retrievers developing HSA being 20%. This breed predisposition suggests a genetic component for HSA [11]. Genome-wide association studies have been carried out to identify risk loci [12, 13]. SNP array analysis of genomic DNA from Golden Retrievers with HSA identified a risk locus on chromosome 5 that was shared by ~ 20% of the cases [12]. In another study, microarray analysis of genomic DNA from dogs with HSA revealed gained copy number aberrations on several genes such as *PDGFRA*, *KDR* and *VEGFA* [13]. A recent whole exome sequencing study of dogs with HSA across a range of breeds revealed somatic mutations in tumor suppressor genes, including *TP53*, and two genes (*PIK3CA* and *PIK3R1*) in the Phosphoinositide 3-kinase (PI3K) pathway [14]. Comparison of somatic copy number aberration profiles in human angiosarcoma and canine hemangiosarcoma identified recurrent copy number gains in *KDR* (31% in human, 22% in canine) and *KIT* (17% in both) [15]. These genome-wide studies reveal that, while specific genetic aberrations are associated with HSA in some populations, these alterations are not sufficient to explain the majority of HSA cases. These observations support the hypothesis that HSA pathogenesis is heterogeneous in nature.

Transcriptome analysis has been previously performed on cell lines and tumor tissues to identify molecular features that define canine HSA. Gene expression profiling of HSA cell lines and non-malignant proliferating endothelial cells revealed that HSA cell lines appeared to overexpress genes associated with inflammation, angiogenesis, adhesion, invasion, metabolism, cell cycle progression, and patterning [16]. Microarray and RNA-seq analysis of visceral HSA tumors identified three distinct molecular subtypes; angiogenesis, inflammation and lipogenesis. These molecular subtypes did not appear to be associated with a specific breed or tumor morphologic subtype [17].

A variety of analytic tools exist for assaying the transcriptome and molecular alterations in tissue. One of these tools, chromatin run-on and sequencing (ChRO-seq), uses RNA polymerase activity to measure transcription and, as such, provides for a highly-sensitive, base-pair level readout of gene expression [18, 19]. Given the lack of clarity regarding the molecular underpinnings of HSA, the major goal of this study was to utilize ChRO-seq to document changes in gene expression between normal splenic tissue and HSA tumor tissue. Our results show that the majority of genes that are upregulated in HSA appear to be associated with extracellular matrix (ECM) remodeling. Additionally, we further characterize two ECM-associated molecules that were highly overexpressed in HSA tumor tissue, podoplanin (PDPN) and laminin alpha 4 (LAMA4). We show by immunohistochemistry (IHC) that the expression of these two cancer-associated factors appears to be primarily limited to HSA tumor cells.

## Results

### ChRO-Seq analysis of transcription in HSA and normal splenic tissue

**Fig. 1:**
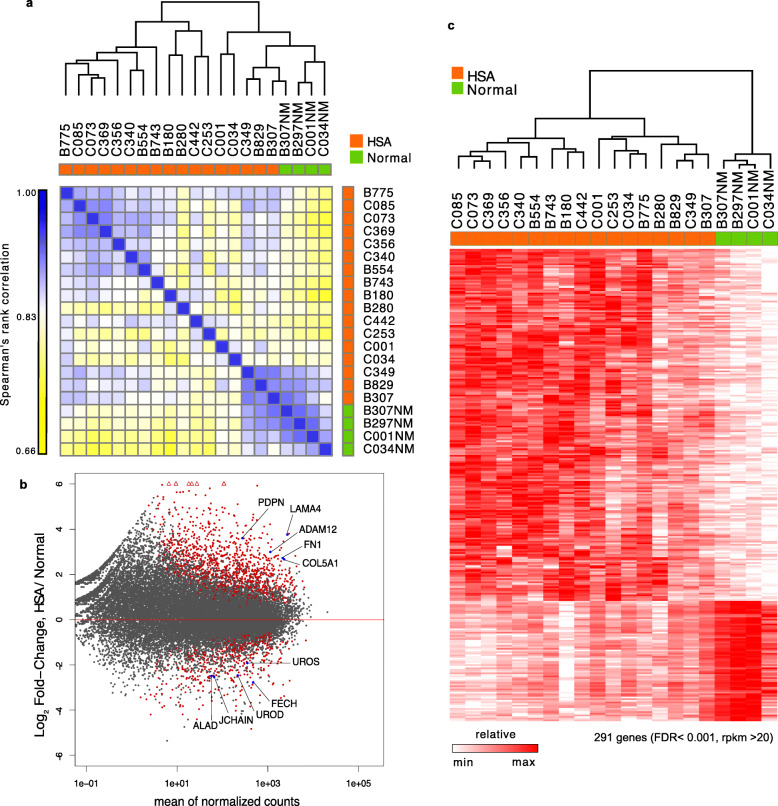
Correlation matrix and MA-plot from ChRO-seq analysis. **a** Spearman’s rank correlation of 17 HSA (orange bar) and 4 Normal samples (green bar). The sample order is based on single-linked hierarchical clustering of the matrix, shown by the dendrogram. 7007 genes (> 20 rpkm) were used to calculate the correlation coefficients by GENE-E. **b** MA-plot of DESeq2 analysis. Results show that ECM-associated gene expression tends to be upregulated in HSA while genes involved in normal splenic function tend to be downregulated. X-axis represents average expression over all samples. Y-axis represents log2 fold change between HSA and normal. Genes with an adjusted *p*-value below 0.01 are shown in red. **c** Heatmap of differentially expressed genes with hierarchical clustering of samples. Differentially expressed genes from DEseq analysis (FDR < 0.001) were examined to evaluate gene expression levels in individual samples

For our genome-wide study, we performed ChRO-seq analysis on tumor tissue from dogs histopathologically diagnosed with HSA (20) and on normal splenic tissue (4) (Table 1). We quantified the similarity of transcription between these tissue samples using Pol II abundance in annotated gene bodies. Three HSA samples (B297, B675, B788) were excluded due to a low number of mappable reads (< 2 million reads). Sequencing data from the remaining samples was then analyzed to create a Spearman’s rank correlation matrix (Fig. 1a). Seven thousand seven genes (> 20 rpkm) were used to calculate the correlation coefficients by GENE-E. Four normal tissues and 3 HSA tissues (B307, B829 and C349) were found to form one cluster, with 2 HSA samples (C001 and C034) also being similar to the Normal/HSA cluster. DESeq2 analysis was then performed on the samples to identify differentially expressed genes. When compared to normal splenic tissue, a total of 906 genes were upregulated in the HSA samples (FDR < 0.005) while 358 genes were downregulated (FDR < 0.005). An MA plot showing the differentially expressed gene set is shown in Fig. 1b. In this plot, the X axis represents the average expression over all samples and the Y axis represents the log2 fold change between the tumor samples and normal spleen. Highly upregulated extracellular matrix (ECM)-associated genes and downregulated heme synthesis-associated genes are labelled with blue dots (Fig. 1b). Differentially expressed genes (FDR < 0.001, 659 genes) were then assessed to evaluate expression levels in individual samples (Fig. 1c). Two hundred and ninety-one genes (rpkm > 20) are shown in the resulting heatmap which also contains an associated hierarchical clustering dendrogram (one minus spearman rank correlation with complete linkage). Results show that the expression profiles of HSA samples appear to be distinct from normal splenic tissue.

**Table 1:** Sample demographics for ChRO-seq, RT-PCR, IHC, and Masson’s trichrome staining

Sample ID	breed	age/sex	mappable reads	RT-PCR	IHC	TC
B775	Std Poodle	9 M	2,896,317			
B297NM	Boxer	8 M	11,511,843		y	
B297^a^		8 M	767,118			
B307NM	Pit Bull	10 F	16,262,534			
B307		10 F	7,465,452			
B675^a^	Lab Ret	10 F	1,445,922			
B788^a^	mixed	unknown F	289,530			
B743	GS	10 F	12,893,170			
B554	mixed	12 M	14,950,537		y	y
B180	GR	11 F	2,398,038			
B280	Lab Cross	11 F	4,095,959			
B829	Flat Coated Ret	9 M	15,142,541			
C073	Lab Ret	12 M	13,668,541			
C085	Bloodhound	12 F	8,272,496	y		
C340	mixed breed	9 M	13,105,709	y		
C349	Ches Bay ret	13 F	6,443,615			
C356	Rhodesian ridge	11 F	12,787,355	y		
C369	mixed breed	11 M	17,736,413			
C001NM	German Shepherd Dog	11 M	38,197,939			
C001		11 M	27,098,934			
C034NM	German Shepherd Dog	11 M	39,192,419			
C034		11 M	19,416,386			
C253	Golden Retriever	11 M	20,874,160			
C442	Golden Retriever	10 M	34,231,915			
B001NM	Beagle	F2	N/A	y		
B004NM	Beagle	F2	N/A	y	y	y
B006NM	Beagle	F2	N/A	y	y	y
B648	Golden Retriever	FS11	N/A		y	y
B172	Boxer	F9	N/A		y	y
B176	Lab Ret	FS13	N/A		y	y
B783	Jack Russell	M14	N/A		y	y
B848	Bulldog	FS12	N/A		y	y

Sample ID (B:CUHA sample, C: CCOGC sample), breed and age/sex are in the table

*NM* normal

^a^sample data were excluded from further analysis due to the low mapped reads

### Extracellular matrix remodeling appears to be a major feature of HSA

**Fig. 2:**
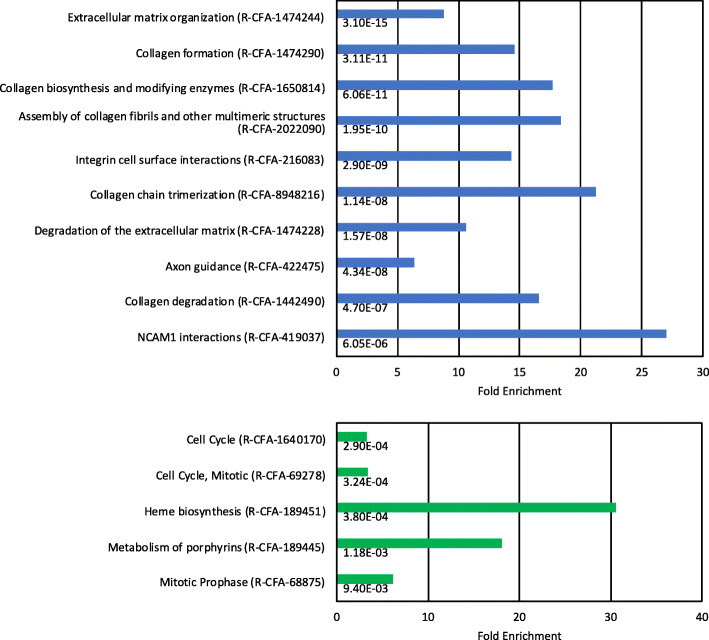
Gene ontology analysis of differentially expressed genes. Upper panel: Top 10 annotation categories identified in upregulated HSA gene set. Lower panel: Annotation categories identified in downregulated HSA gene set. X axis represents fold enrichment, FDR values are labelled under each bar

When evaluating the top 50 most highly upregulated genes in the HSA group, we observed that many of these molecules are associated with the ECM, examples of which include podoplanin (PDPN), laminin alpha 4 (LAMA4), ADAM12, and fibronectin 1 (Fig. 1b). To test the prevalence of ECM-associated molecules in our dataset, we carried out gene ontology (GO) analysis using PANTHER (protein analysis through evolutionary relationships) (Fig. 2). We utilized a total of 369 of the most highly upregulated genes (FDR < 0.0005) as an input list, with 282 of these genes then being identified as uniquely mapped genes in a *Canis lupus familiaris* reference database. A total of 20 annotation categories were detected (FDR < 0.05) from the reactome pathway database, with the top 10 annotation categories from this dataset being found to be related to ECM regulation. A more detailed list of the ECM-associated factors that were upregulated in HSA tumor tissue is shown in Table 2, along with their fold change (log_2_) and FDR. A full list of Reactome pathways terms and the list of genes is shown in the supplemental table.

**Table 2:** List of upregulated genes related to ECM

ECM proteins		log2 fold change	FDR
COL5A3	Collagen type V alpha 3 chain	4.1598	6.87E-06
COL18A1	Collagen type XVIII alpha 1 chain	3.5531	5.90E-06
COL15A1	Collagen type XV alpha 1 chain	3.4661	3.79E-06
COL1A1	Collagen alpha-1(I) chain	3.2225	7.54E-05
COL3A1	Collagen type III alpha 1 chain	3.1839	2.31E-05
COL16A1	Collagen type XVI alpha 1 chain	2.8378	1.37E-05
COL27A1	Collagen type XXVII alpha 1 chain	2.6061	0.0001001
COL5A2	Collagen type V alpha 2 chain	2.5905	1.21E-05
COL6A3	Collagen type VI alpha 3 chain	2.4047	0.00016717
COL6A2	Collagen type VI alpha 2 chain	2.3539	0.00022812
COL24A1	Collagen type XXIV alpha 1 chain	2.1612	9.72E-05
COL6A1	Collagen type VI alpha 1 chain	2.1272	0.0003894
COL4A2	Collagen type IV alpha 2 chain	1.7088	0.00033908
POSTN	Periostin	6.7428	1.01E-07
SPP1	Secreted phosphoprotein 1;Osteopontin	4.6241	2.73E-05
**LAMA4**	**Laminin subunit alpha 4**	**3.7745**	**1.58E-09**
LAMB4	laminin subunit beta-4	2.1244	4.93E-05
FN1	Fibronectin	2.7327	2.75E-06
LAMC2	Laminin subunit gamma 2	2.4019	0.00041187
ECM binding protein		log2 fold change	FDR
LUM	Lumican	4.9497	0.00013751
ITGA2	Integrin subunit alpha 2	3.1184	0.00031867
ITGA6	Integrin subunit alpha 6	2.3456	4.69E-05
DDR2	Discoidin domain receptor tyrosine kinase 2	2.1874	0.00011485
BGN	Biglycan	2.1703	0.00022031
ITGA5	Integrin subunit alpha 5	1.9946	1.61E-06
PDPN*	**Podoplanin**	**3.6171**	**6.43E-05**
ECM related enzyme		log2 fold change	FDR
ADAMTS14	ADAM metallopeptidase with thrombospondin type 1 motif 14	3.5027	1.30E-08
ADAM12	ADAM metallopeptidase domain 12	3.0033	1.90E-10
TLL1	Metalloendopeptidase	3.2116	0.00023572
ADAMTS4	ADAM metallopeptidase with thrombospondin type 1 motif 4	2.7526	0.00018318
TIMP1	Metalloproteinase inhibitor 1	2.4316	0.00021979
P4HA2	Prolyl 4-hydroxylase subunit alpha 2	1.7259	2.73E-06
PLOD1	Procollagen-lysine,2-oxoglutarate 5-dioxtgenase 1	1.6827	0.0001534
MMP14	Matrix metalloproteinase	1.6784	6.43E-07

Interestingly, when we queried genes in the downregulated dataset (124 genes with an FDR cut-off of 0.0005), we did not observe statistically significant functional associations using GO analysis. Therefore, we adjusted the FDR cut-off to 0.005 and this adjustment identified 358 genes as being downregulated in the HSA samples with 301 of these genes being uniquely mapped in the reference database. As shown in Fig. 2, five annotation categories were detected in this dataset, with the most enriched reactome pathway term being heme biosynthesis. Examples of specific genes that are associated with heme biosynthesis include, UROS, FECH, UROD, JCHAIN, and ALAD (Fig. 1b). This result suggests that the expression of genes involved in normal splenic function is suppressed in HSA tumor tissue.

### Characterization of ECM-associated proteins in HSA tumor tissue

**Fig. 3:**
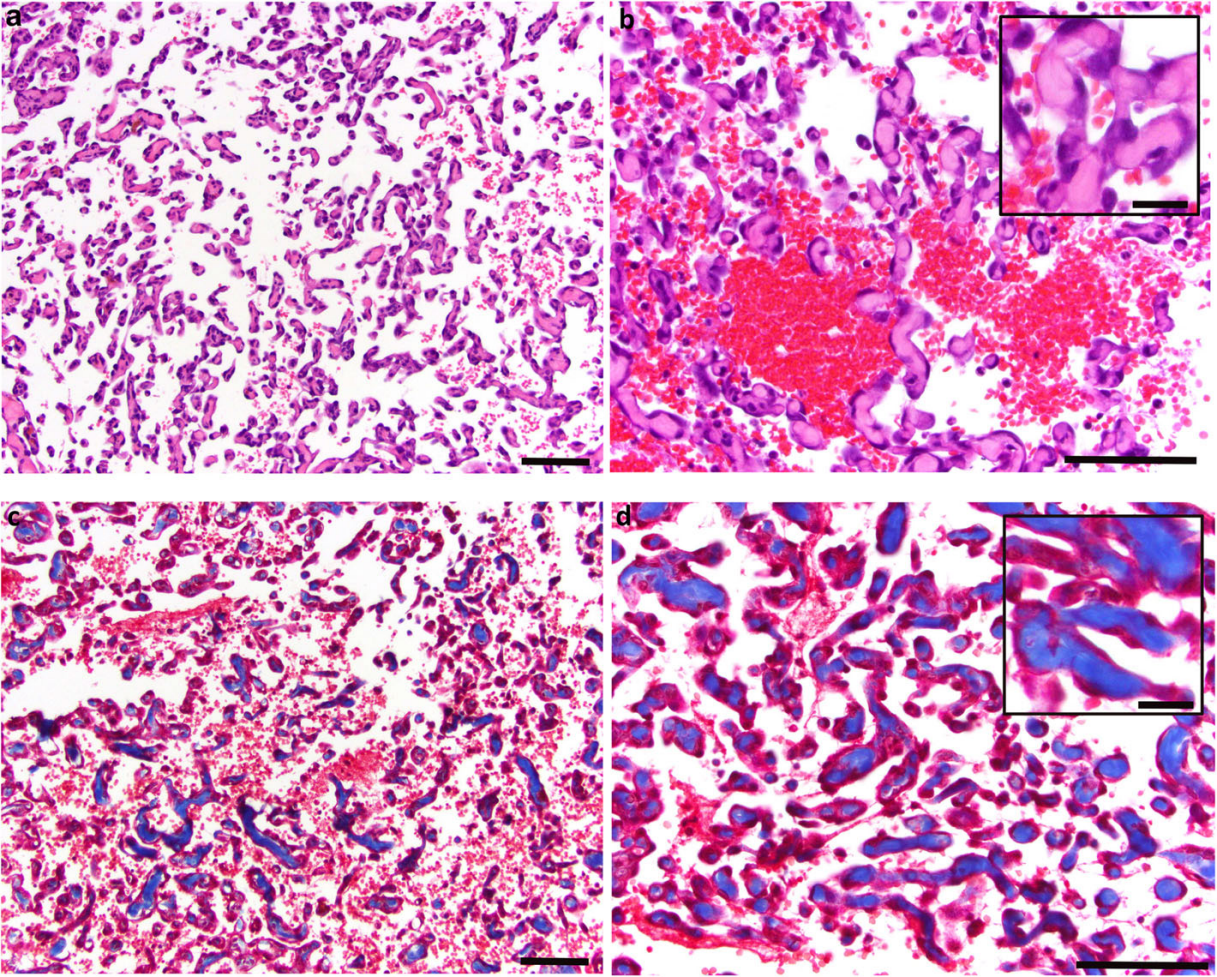
Histology of H&E (top) and Masson’s trichrome (bottom)-stained HSA tumor sections. H&E staining shows that collagen bundles are surrounded by neoplastic endothelial cells and appear to form “inside out” blood vessels (**a** and **b**). Trichrome-stained HSA sections (**c** and **d**) show collagen bundles (blue staining) in vascular regions of the tumor are surrounded by endothelial cells. **a** and **c**: low magnification, (b and d): high magnification. Scale bars represent 50 μm in a-d, and 10 μm in insets in **b** and **d**. HSA (B783) FFPE sections were stained

To more directly test the hypothesis that ECM-associated factors are overexpressed in HSA tumor tissue, we first stained paraffin sections of tumor and normal tissue with Masson’s trichrome to label collagen fibers (Fig. 3). Staining of the more vascular regions of the HSA tumor tissue show that collagen fibrils are abundant throughout these regions. This observation fits well with our finding that multiple collagen isoforms were highly overrepresented in our HSA-overexpression ChRO-Seq dataset. Interestingly, in these vascular regions, the malignant tumor cells appear to surround the collagen bundles, forming what resembles inverted blood vessels. To further support these findings, images and quantitative data from six additional HSA and two normal splenic samples are shown in supplemental figure, S1.

**Fig. 4:**
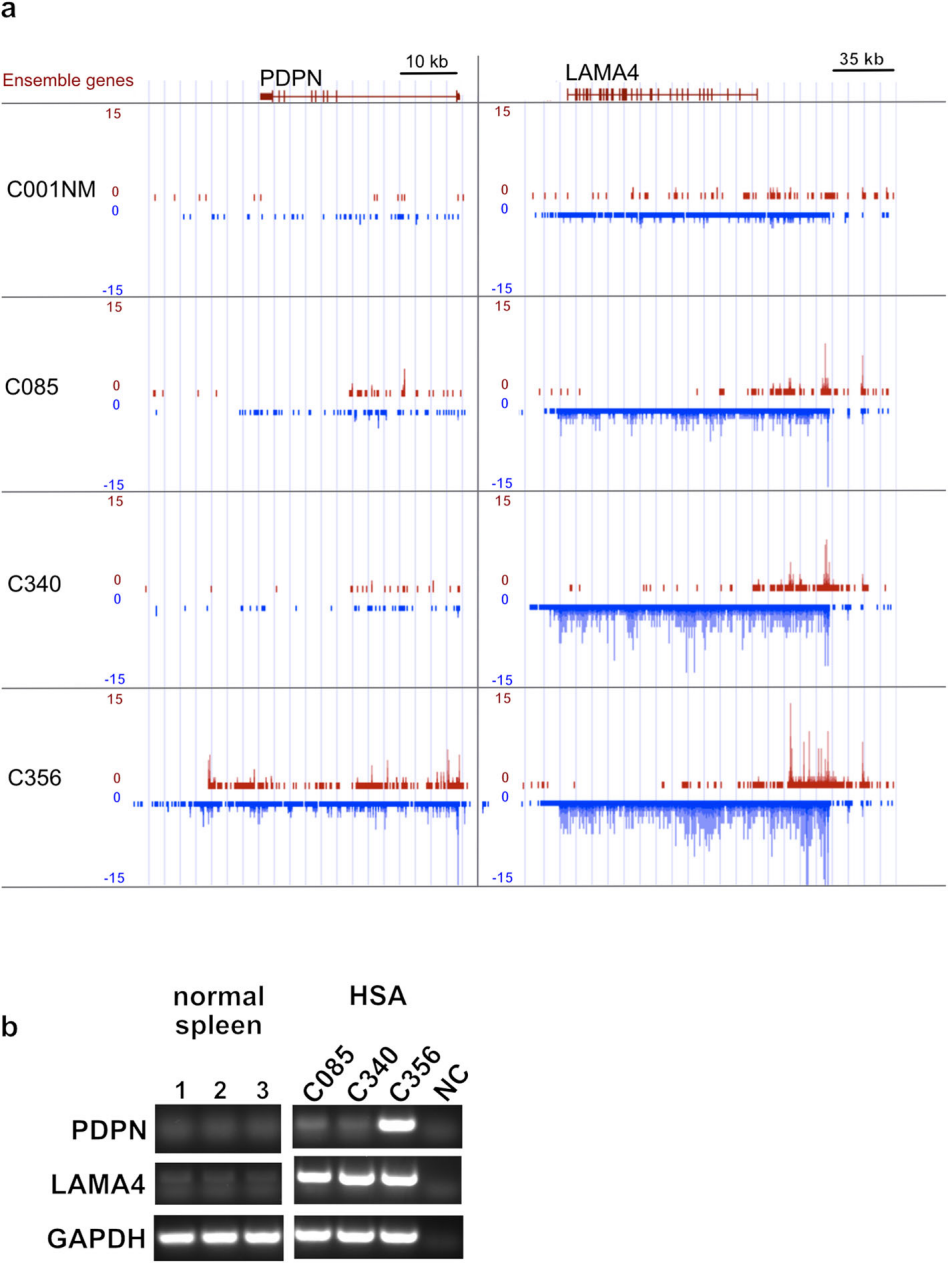
PDPN and LAMA4 transcription and mRNA expression in HSA and normal splenic samples. **a** Genome browser views (UCSC browser) of the ChRO-seq dataset showing PDPN and LAMA4 gene loci. ChRO-seq reads on sense and anti-sense strands are shown in red and blue, respectively. Red lines indicate the encoding gene in the Ensemble database. **b** RT-PCR analysis documenting PDPN and LAMA4 transcript expression in normal (1: B001NM, 2: B004NM, 3: B006NM) and HSA samples corresponding to ChRO-seq data (C085, C340 and C356). GAPDH primers were used as a positive control. Negative control (NC) lacks cDNA template. Results show that PDPN and LAMA4 mRNA do not appear to be expressed in normal spleen. LAMA4 transcripts were observed in all three HSA samples while PDPN showed strong expression in one of the three HSA samples

We next further characterized two ECM-associated factors that were highly overexpressed in HSA tumor tissue. For this analysis we focused on PDPN (FDR 6.43E-05, fold change 3.62) and LAMA4 (FDR 1.58E-09, fold change 3.77) because these molecules have been previously associated with cancer progression in other tumor types [20–25]. For further characterization, we first investigated PDPN and LAMA4 expression levels in our ChRO-seq dataset. A genome browser view of transcription levels from one normal and three HSA samples is shown in Fig. 4a. Expression levels from all ChRO-seq samples are shown in supplemental figure S2. Results show that, at the PDPN locus, two of the HSA cases (C085 and C356) showed increased RNA polymerase activity compared to normal tissue, while expression levels of the third HSA sample (C340) was comparable with normal tissue. LAMA4 was transcribed from the minus strand and all HSA samples showed higher transcriptional activity than normal tissue. We next investigated the expression of mature PDPN and LAMA4 transcripts in tumor tissue and normal spleen using RT-PCR. Results show that, while PDPN or LAMA4 transcripts were either absent or weakly expressed in normal spleen, LAMA4 was significantly expressed in all three tested HSA tumor samples while PDPN was robustly expressed in one (C356) of the three HSA samples (Fig. 4b and supplemental Figure S7).

**Fig. 5:**
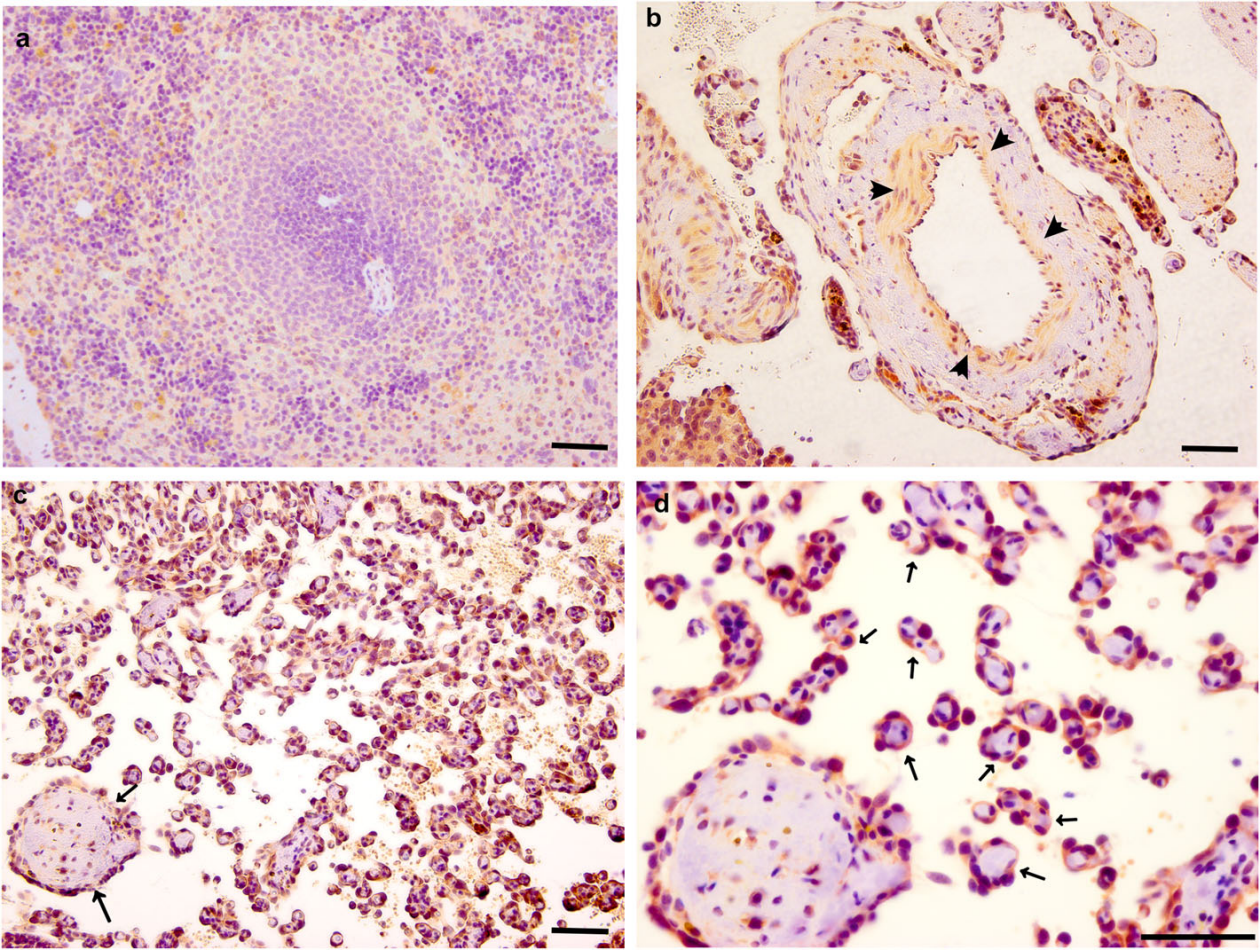
Immunohistochemical localization of PDPN in HSA and normal splenic samples. **a** PDPN detection in normal splenic tissue was low to absent (B297, normal white pulp). **b** anti-PDPN staining appears weak in endothelial cells of blood vessels that are adjacent to tumor masses (B783). **c** and **d** anti-PDPN staining is strong in cells within the tumor’s vascular regions (B848). In these regions the PDPN-positive tumor cells were seen to surround what appears to be collagen bundles. PDPN was also detected in tumor cells from the more avascular regions of the tumor (Supplemental materials). c = low magnification, d = high magnification. Scale bar represents 50 μm

**Fig. 6:**
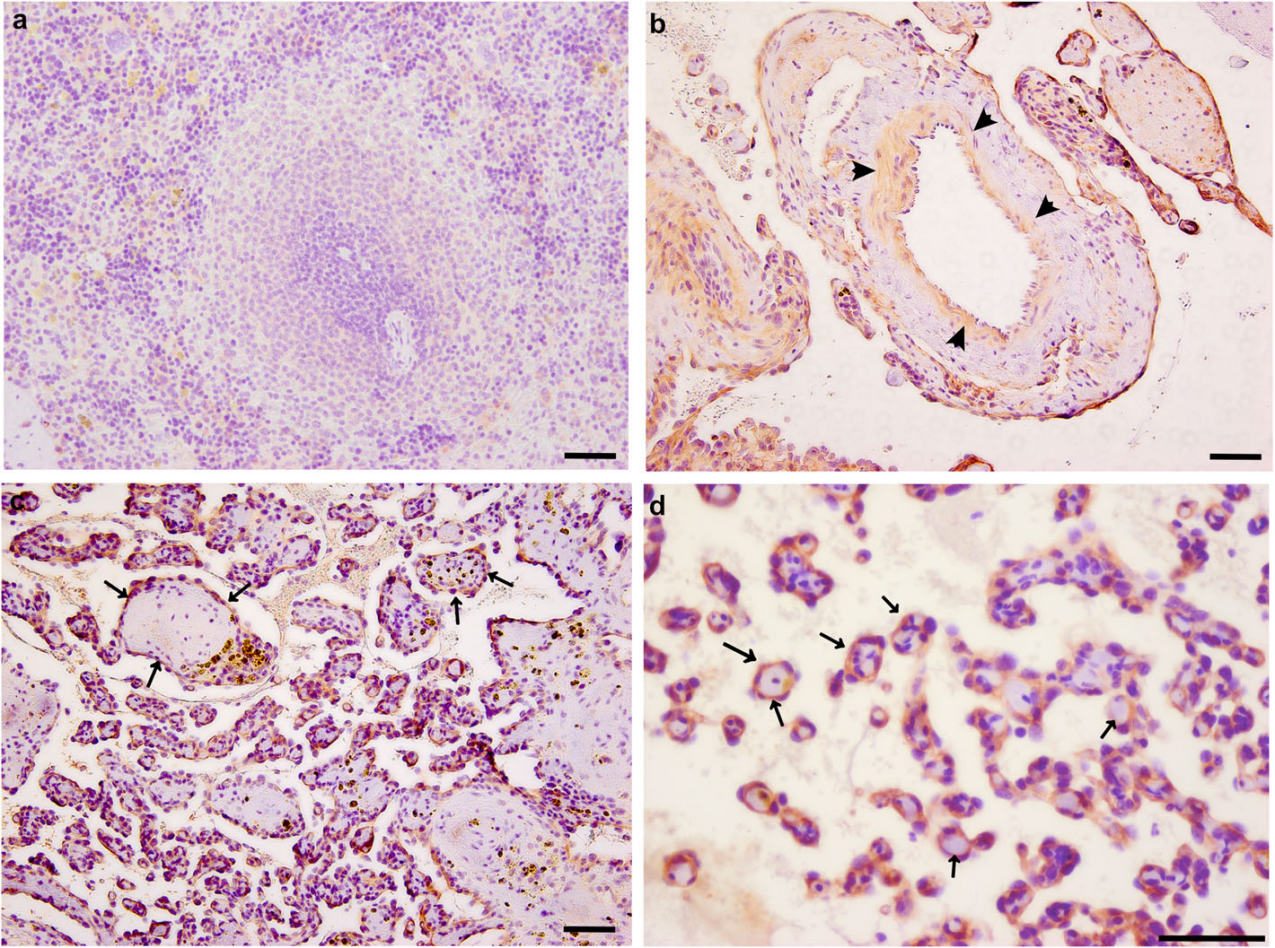
Immunohistochemical localization of LAMA4 protein in HSA and normal splenic samples. **a** LAMA4 detection was low to absent in normal splenic tissue (B297, normal white pulp). **b** anti-LAMA4 staining appears weak in endothelial cells of blood vessels that are adjacent to tumor masses (B783). **c** and **d** anti-LAMA4 staining is strong in tumor cells within the tumor’s vascular regions (B848). In these regions the LAMA4-positive tumor cells are seen to surround what appears to be collagen bundles. LAMA4 was also detected in tumor cells from the more avascular regions of the tumor (Supplemental materials). c = low magnification, d = high magnification. Scale bar represents 50 μm

Next, we performed IHC analysis of PDPN and LAMA4 on HSA tumor sections to confirm that these molecules are expressed in tumor tissue at the protein level and to investigate their localization. HSA samples B297, B783 and B848 are shown in Figs. 5 and 6 with images and quantitative data from additional samples being shown in supplemental Figure S3 and S4. IHC analysis found that anti-PDPN staining in normal splenic tissue was low to absent. In HSA tumor tissue, however, anti-PDPN staining was robust in the cytoplasm of tumor cells encircling the collagen fibrils (Fig. 5c and d, arrow). In addition to tumor cells, anti-PDPN staining was also evident in the cytoplasm of endothelial cells in tumor-adjacent blood vessels (Fig. 5b, arrowhead). The localization pattern of anti-LAMA4 staining was similar to that of PDPN, with little staining being seen in normal splenic tissue and strong anti-LAMA4 staining being observed in the cytoplasm of HSA tumor cells encircling collagen fibrils. Again, as with PDPN, anti-LAMA4 staining was also observed in the cytoplasm in the endothelial cells from tumor-adjacent blood vessels (Fig. 6c and d).

Serial sections of additional HSA samples were then stained with Masson’s trichrome and immunohistochemistry was performed on these additional tissues using anti-PDPN and LAMA4 antibodies (Supplemental figure S5 and S6). Sections from one sample (B176, Figure S5) showed solid/cavernous features with blood vessels (Figure S5 a). Masson’s trichrome staining highlighted the presence of collagen fibrils filling spaces between regions of neoplastic cells (Figure S5 a and b). Anti-PDPN staining appeared to be primarily cytosolic in these neoplastic cells and in endothelial cells lining adjacent blood vessels (Figure S5 c). Anti-LAMA4 staining was strong in extracellular regions of the tumor and in endothelial cells lining blood vessels (Figure S5 d). Sections from sample B554 primarily showed capillary/cavernous features (Figure S6). Masson’s trichrome staining of this sample revealed a core collagen region surrounded by malignant endothelial cells with spindle shaped nuclei (Figure S6 a and b). Anti-PDPN and LAMA4 staining appeared to be primarily cytosolic in tumor cells from this sample.

## Discussion

In this study, we first utilized ChRO-seq to identify RNA polymerase activity in hemangiosarcoma tumor tissue and normal splenic tissue. Analysis of the correlation matrix for our ChRO-seq dataset finds that 14 of the HSA tumor samples clustered together, while 3 of the HSA samples appeared to be more similar to normal splenic tissue (Fig. 1). While speculative, it is possible that these 3 samples represent a different subtype of HSA and, as such, displayed a gene expression profile that is more similar to normal tissue. Alternatively, it is also possible that these 3 presumptive HSA tissues were actually tumor-adjacent normal tissue or a mix of normal and tumor tissue. While grading of canine hemangiosarcoma is not often utilized due to the aggressive nature of the neoplasm, a grading scheme does exist and differences in gene expression between the samples may correlate with progression and eventual outcome [26].

Previous HSA transcriptomic profiling studies have implicated a number of signaling pathways in HSA pathogenesis. Tamburini et al., for example, found that, when compared to cell lines derived from splenic hematomas, HSA cell lines exhibited several different distinct gene expression profiles, including signatures for angiogenesis, inflammation, adhesion, invasion metabolism, cell cycle and signaling. In another study, Gorden et al. performed microarray and RNA-seq analyses on visceral HSA tumor tissues (12 spleen, 7 heart, 4 liver, 1 lung) and identified three distinct tumor subtypes that were associated with either angiogenesis, inflammation, and adipogenesis. Our finding that ECM remodeling appears to be a major gene expression signature in HSA differs from these previous studies. One possible reason for the difference in outcomes between our study and the Tamburini study is that our report compared gene expression patterns in HSA splenic tissue with normal splenic tissue while the Tamburini study was comparing the gene expression profiles of HSA cell lines with splenic hematoma cell lines [16]. Thus, the differences may be due to the fact that the expression profile of normal splenic samples differs significantly from hematoma samples. Additionally, these differences may also be because the expression profile of cell lines may differ significantly from primary tissue samples due to decreased cellular complexity and prolonged cell culture. One potential reason why outcomes from our study differed from the Gorden study is that we evaluated differences in gene expression patterns between HSA and normal splenic tissue while the other study analyzed gene expression patterns within HSA tissues. Lastly, another reason why outcomes from our study may have differed from both of these previous studies is that ChRO-seq analysis detects nascently synthesized RNA as opposed to mature transcripts, whose levels can be affected by a variety of factors including transcript stability.

Regarding the specific types of ECM factors that were upregulated in our dataset (Table 2), we identified genes encoding fibrous components of the ECM, including 12 collagen genes, 3 laminin genes, and fibronectin. Several ECM binding proteins were also identified in this dataset including, Lumican and Biglycan, which are small leucine-rich proteoglycans that regulate collagen fibril and matrix assembly [27–30]. Integrin alpha 2 is directly associated with fibril-forming collagens (1,2,3,5,6,14, 18) [31] while DDR2 functions as a cell surface receptor for fibrillar collagen and regulates cell differentiation by remodeling ECM [32, 33]. Additionally, integrin alpha 5 binds directly to fibronectin while integrin alpha 6 binds to laminin [34, 35]. Several ECM-related enzymes were also found in this dataset including P4HA2 and PLOD1 which catalyze collagen biosynthesis [36, 37] along with ADAMTS14, ADAMTS4, ADAM12 and TLL1 which process procollagen to collagen by cleaving N-propeptide and C- propeptide [38, 39]. Lastly, two molecules directly involved in ECM turnover, tissue inhibitor of metalloproteinase 1 (TIMP1) and matrix metalloproteinase 14 (MMP14), were also found in this dataset [40–42].

The extent to which ECM genes were upregulated in HSA tumors in our study is highlighted by our GO analysis which found that 9 of the top 10 biological process categories were ECM-related (Fig. 2). Interestingly, 5 of these 9 categories related to collagen function. We more directly tested for the abundance of collagen fibers in the tumor samples using Masson’s trichrome stain and found extensive collagen deposition throughout the tumor tissue (Fig. 3). Collagen deposition was observed both in the more solid areas of the tumor and, in particular, in the tumor regions filled with malformed vascular channels. In these vascular regions, neoplastic endothelial cells are often found to encircle the collagen bundles forming “gumball”-shaped structures that look like inverted blood vessels. While stromal fibroblasts are presumably primarily responsible for the synthesis of these collagen fibers, it is also possible that the tumor cells may also be partly responsible for synthesis of these collagen fibers.

In addition to collagen, we also found that PDPN and LAMA4 were highly overexpressed in HSA tumor tissue when compared to normal tissue. These molecules were of particular interest to us given their close association with cancer progression in other types of cancer. Podoplanin is a mucin-type protein that contains an extracellular region, transmembrane domain, and intracellular tail. It is widely known as a marker for lymphatic endothelial cells and also to play a critical role in heart and lung development and in development of the lymphatic endothelial system [43–45]. PDPN appears to play several roles in cancer progression. A number of studies have shown that PDPN expression in cancer cells promotes tumor cell proliferation and invasion [20–22]. In addition to cell-intrinsic effects, PDPN is also believed to promote tumor metastasis by interacting with its receptor, CLEC2, on the platelets. This interaction then promotes the coating of tumor cells by platelets, thereby protecting tumor cells from the immune system [46]. In addition to its role in human cancers, PDPN is also overexpressed in canine squamous cell carcinomas and melanomas [47] and PDPN mAbs were recently found to have potent anti-tumor activity in mouse xenograft models of canine melanoma [48]. Interestingly, overexpression of PDPN in mice leads to disseminated intravascular coagulation [49], a condition that is strikingly similar to that seen in many dogs with HSA [50]. Our ChRO-seq data demonstrated that PDPN expression varies significantly between HSA samples (Figs. 1 and 4). IHC analysis found that PDPN was robustly expressed in transformed endothelial cells in certain HSA tumor samples. However, similar to our ChRO-seq finding, we did find that PDPN appears to only be expressed in a subset of HSA tumors. In future studies, we plan to test whether PDPN expression in HSA tumors correlates with disease severity.

Laminins form large heterotrimeric αβγ protein complexes and are a prominent component of basement membranes. LAMA4 is distinct from other laminin isoforms in that it lacks a polymerization domain with the loss of this domain potentially facilitating tumor cell migration [23]. Interestingly, LAMA4 was recently described as an “oncolaminin” due to its strong association with cancer cell migration and tumor progression in a range of cancers [24]. These links to cancer include recent studies which found that LAMA4 and MCAM (melanoma cell adhesion molecule) are highly enriched in tumor blood vessels in renal cell carcinoma and colorectal carcinoma. Additionally another study found that expression of these molecules is enhanced in locally invasive and metastatic clear cell renal cell carcinoma [25]. Further, antisense oligonucleotides against laminin-8 (LAMA4 and LAMB1) were found to block the invasion of glioma cells and neovascularization in vitro [51]. Taken together, these published studies indicate that LAMA4 plays a key role for vascular development, tumor progression and metastasis. In our study, deseq2 analysis found that LAMA4 is highly transcribed in HSA tumor tissue. This observation was supported by PCR analysis of mRNA isolated from HSA tumor tissue (Fig. 4 and supplemental Figure S7). Additionally, our IHC analysis found that LAMA4 protein expression appears to be primarily limited to malignant endothelial cells (Fig. 5). Given the previously defined roles for LAMA4 in cancer progression and tumor metastasis, our findings raise the possibility that LAMA4 is an important mediator of canine HSA pathobiology.

## Conclusions

Outcomes from our studies found that the majority of upregulated genes in HSA tumor tissue appear to be associated with ECM remodeling. This finding was supported by IHC analysis which found a robust collagen network throughout HSA tumor tissue. Additionally, we further characterized two ECM-associated factors, PDPN and LAMA4, and found that their expression was largely limited to tumor cells in HSA tissue. Both of these molecules have been previously identified as potential biomarkers in other types of cancer. Given the recent previous preclinical mouse studies showing that anti-canine PDPN antibodies can block the growth of melanomas, our findings raise the possibility that similar types of therapies may have utility for treating canine HSA.

## Methods

### Samples

Canine tissue samples were obtained from the Canine Comparative Oncology & Genomics Consortium and Cornell University Hospital for Animals. Pathology was independently confirmed, and patient demographics are described in the supplemental material (Table 1). ChRO-seq libraries were made from splenic hemangiosarcoma (*n* = 20) and normal splenic tissues from HSA dogs (*n* = 4). Normal splenic samples from three beagles were either snap-frozen or fixed in PFA and Paraffin-embedded (FFPE) for sectioning. Additional FFPE sections of HSA cases were obtained from Cornell University Hospital for Animals.

### Chromatin- run-on and sequencing

Chromatin was extracted from each tissue sample and chromatin run-on was performed as described previously [18, 19, 52]. ChRO-seq library preparations were executed according to the Illumina protocol and were sequenced using Illumina NextSeq500 sequencing. Raw sequence FASTQ files were assessed by FastQC for quality control [53]. Single-read sequencing data were preprocessed and mapped to the canine genome assembly using the proseq 2.0 pipeline (https://github.com/Danko-Lab/proseq2.0) [54]. Briefly, Single-read sequencing data were preprocessed to remove the adapter sequences and trimmed based on base quality, and deduplicating the reads based on unique molecular identifiers in RNA adapters. Sequencing reads were mapped into a canine genome assembly (CanFam 3.1) using the Burrows-Wheeler Aligner software package [55]. The number of mappable reads are listed in Table 1. Aligned BAM files were converted into bigWig format, which were used to create the matrix of read counts mapping to each annotated gene (CanFam3 ensGene). Seven thousand seven genes (> 20 rpkm) were used to calculate the Spearman’s rank correlation coefficients to create the matrix and dendrogram by GENE-E (https://software.broadinstitute.org/GENE-E/). The list of differentially expressed genes and an MA-plot were produced by running DESeq2 (false discovery rate < 0.01) [56]. Upregulated genes (369 genes, FDR < 0.0005) and down regulated genes (358 genes, FDR < 0.005) in HSA were subjected to PANTHER (Protein Analysis Through Evolutionary Relationships) overrepresentation gene ontogeny analysis (GO) on Reactome pathway dataset (version 65, released 2019-12-22) [57]. Differentially expressed genes (FDR < 0.001, 659 genes) were assessed to show the expression level in individual samples. Heatmaps and dendrograms were created by GENE-E.

### Immunohistochemistry and histology staining

Prior to all staining protocols, paraffin section slides were rehydrated in xylene followed by sequential washes in 100, 90, 80, and 70% EtOH. H&E and Masson’s trichrome staining were performed on 10 HSA samples and 4 normal spleen sections. Gill’s hematoxylin and Eosin Y solutions were used for H&E. Masson’s trichrome staining was performed following the manufacture’s protocol (Masson’s Trichrome Stain Kit, Polysciences, Inc. #25088–100). Immunohistochemistry (IHC) experiments using anti-canine PDPN (clone PMab-38 [58], now commercially available at FUJIFILM Wako Pure Chemical Corporation, Osaka, Japan; 017–27,091, 1:200) and anti-LAMA4 (Sigma-Aldrich Corp. St. Louis, MO, HPA015693, Human Atlas antibodies for IHC, Rabbit polyclonal, epitope homology 85%, 1:300), were performed on 8 HSA samples and 2 normal spleens following a standard protocol [59]. Briefly, slides were boiled for 40 min in 0.01 M sodium citrate to retrieve antigens. Slides were cooled and washed in running tap water for 20 min, then incubated for 15 min in 3% hydrogen peroxide to quench endogenous peroxidases. Slides were blocked in 2.5% normal goat serum for 30 min at room temperature. Primary antibodies were diluted in 1% BSA/TBST, and incubated overnight at 4C. Slides were washed three times with TBS-T, then incubated with a secondary antibody for 1 h at room temperature (Goat anti-mouse-HRP, Vector laboratories, Inc., Burlingame, CA; #MP7452 or Goat anti-rabbit-HRP, Vector laboratories, Inc., #MP7451). ImmPACT NovaRED peroxidase substrate (Vector laboratories, Inc., SK-4805) with hematoxylin counterstain was used for signal detection. Slides were dehydrated through 80, 95 and 100% EtOH to Xylene and coverslipped. Blue staining and NovaRED signals were quantified using image J (see supplemental method and figures S1-S7).

### RT-PCR

Snap frozen tissues (3 normal spleens and 3 HSA tissues, see Table 1 for demographics) were pulverized in a tissue crusher and total RNA was extracted with Trizol reagent. 1 μg of RNA was reverse-transcribed to cDNA using High-capacity cDNA reverse transcription kit (Applied Biosystems) following the manufacture’s protocol (25 °C for 10 min, 37 °C for 120 min, 85 °C for 5 min). All cDNA reactions were diluted 5-fold with water prior to use in PCR. 1 μl of diluted cDNA was used for PCR using Go-Taq master mix (Promega Corporation) with canine specific primers (0.4 μM each, sequences are shown in supplemental material), products were amplified using the following parameters: 95 °C for 5 min, 30 cycles of 3 steps (95 °C for 30 s, 58 °C for 30 s, and 72 °C for 20 s) and 72 °C for 5 min. The amplified products were analyzed using agarose gel electrophoresis (1.2%).

## Supplementary information

**Additional file 1.** Supplemental methods and figure legends.

**Additional file 2.** Supplemental tables for gene ontology analysis.

**Additional file 3.** FS1. Trichrome staining quantification.

**Additional file 4.** FS2. ChRO-seq counts on PDPN and LAMA4 gene bodies.

**Additional file 5.** FS3. PDPN immunohistochemistry quantification.

**Additional file 6. **FS4. LAMA4 immunohistochemistry quantification.

**Additional file 7. **FS5. Masson’s trichrome staining and IHC on serial sections from case B176 (HSA).

**Additional file 8. **FS6. Masson’s trichrome staining and IHC on serial sections from case B554 (HSA).

**Additional file 9. **FS7. Original gel images for Fig. 4b.

## Data Availability

The raw files (FASTQ) and processed files (BigWig) analysed during the current study are available in the Gene Expression Omnibus GSE150705 (https://www.ncbi.nlm.nih.gov/geo/query/acc.cgi?acc=GSE150705).

## Abbreviations

HSA: Hemangiosarcoma
ChRO-seq: Chromatin run-on sequencing
IHC: Immunohistochemistry
ECM: Extracellular matrix
PDPN: Podoplanin
LAMA4: Laminin alpha 4
AS: Angiosarcoma
PI3K: Phosphoinositide 3-kinase
GO: Gene ontology
PANTHER: Protein analysis through evolutionary relationships
FFPE: Fixed in PFA and Paraffin-embedded
RT-PCR: Reverse transcription – polymerase chain reaction
