# Supplementary material for: Chromatin run-on sequencing analysis finds that ECM remodeling plays an important role in canine hemangiosarcoma pathogenesis
Source: BMC Vet Res. 2020 Jun 22;16:206. doi: 10.1186/s12917-020-02395-3 (PMC7310061; doi:10.1186/s12917-020-02395-3)
Supplement: Supplementary file 1 — Additional file 1. Supplemental methods and figure legends. [file 12917_2020_2395_MOESM1_ESM.pdf]

Methods for Supplemental figures

Quantitative analysis of Masson's trichrome and immunohistochemical staining using ImageJ.
For quantification of Masson's trichrome staining, all images were taken using x20 objectives and adjusted to the resolution of 5.84 pixel/um in ImageJ. An RGB stack was obtained and threshold was adjusted to distinguish blue signal from blank area using the blue channel (min/max range, 135-185/181-232). The area of blue signal that was limited to the threshold value was measured in μm^2 and a bar graph of the area was created. Note that color intensity was not measured.

For quantification of immunohistochemical staining, all images were taken using x20 objectives and adjusted to a resolution of 5.84 pixel/um in ImageJ. Color deconvolution was performed using the Hematoxylin-DAB mode, and the threshold was adjusted to distinguish brown signal from blank area in channel 2 (DAB, min/max range, 0-119/126-169). The area of brown signal was measured in μm^2 and a bar graph of the area was created. Note that intensity of color was not measured. Signals from anti-PDPN and LAMA4 staining were quantified in the cytosolic and extracellular areas and, therefore, signal normalization based on the number of cells was not performed.

Primers for RT-PCR (5' ->3')
These canine specific primers were selected by using the Primer-BLAST program (https://www.ncbi.nlm.nih.gov/tools/primer-blast/).
GAPDH (F: TGTCCCCACCCCCAATGTATC, R: CTCCGATGCCTGCTTCACTACCTT),
PDPN (F: TAACGTGGTGACTGACGGTG, R: AGTCGGCCCATCCTCTAAGT),
LAMA4 (F: TGACACCCGCTCTCTGTTTC, R: CCTTCCACCTGTTCCCTCAC).

Supplemental tables for gene ontology analysis (excel file)
Excel file contains 5 sheets.

1. File contents
2. Gene ontology results from upregulated genes
3. Gene ontology results from downregulated genes
4. List of upregulated genes from DESeq2
5. List of downregulated genes from DESeq2

Supplemental figure legends
Supplemental Figure S1. Trichrome staining quantification
The blue areas of Masson's trichrome staining were quantified using image J. Two images were taken for each case, including spleens from 2 normal dogs and spleens from 6 dogs with HSA (please see Table 1 for sample demographics).

Supplemental figure S2. ChRO-seq counts from PDPN and LAMA4 gene bodies.

Supplemental figure S3. Quantification of anti-PDPN staining.
NovaRed signal (brown) from each slide was quantified using image J. Serial sections from HSA sample, B648, were used as a no primary control and for anti-PDPN staining (3A). Two images

were taken for each case and a total of 6 HSA cases were examined (3B). Images from cases B783 and B848 are also shown in Figure 5 of the main text.

Supplemental Figure S4. Quantification of anti-LAMA4 staining. NovaRed signal (brown) on each slide was quantified using image J. Serial sections from the B648 HSA sample were used for the no primary control group and the anti-LAMA4 group (4A). Two images were taken for each case and a total of 6 HSA cases were examined (4B). Images from the B783 and B848 samples were also shown in the main text (Figure 6).

Supplemental Figure S5.
Masson's trichrome staining and IHC analysis of serial sections from HSA case (B176). This HSA lesion shows solid/cavernous features with blood vessels (H&E). Masson's trichrome staining highlighted the distribution of collagen (blue staining) in the spaces (green arrows in b) between neoplastic cells which often contain a round-oval shape nucleus with mitotic figures (green arrows in a). PDPN signal appears to primarily be localized to cytosolic regions in the neoplastic cells (black arrows in c) and in endothelial cells from nearby blood vessels (circle arrows in c). LAMA4 signal appears to be strong in extracellular regions (black arrows in d) as well as in endothelial cells from blood vessels (circle arrows in d). Scale bar: 50 µm.

Supplemental Figure S6.
Masson's trichrome staining and IHC of serial sections from HSA sample (B554). This HSA lesion shows capillary/cavernous features (H&E). Masson's trichrome staining allowed for visualization of a collagen core (blue staining) surrounded by malignant endothelial cells with spindle shape nuclei (green arrows in a). PDPN and LAMA4 signals appear to be primarily cytosolic in these malignant endothelial cells (black arrows in c and d). Neoplastic cells with oval shaped nuclei also contain some staining in the cytosol (circle arrows in c and d). Scale bar: 50 µm.

Supplemental Figure S7. Original gel images for Figure 4b.
