# Supplementary figures and images for: Chromatin run-on sequencing analysis finds that ECM remodeling plays an important role in canine hemangiosarcoma pathogenesis
Source: BMC Vet Res. 2020 Jun 22;16:206. doi: 10.1186/s12917-020-02395-3 (PMC7310061; doi:10.1186/s12917-020-02395-3)

Supplemental Figure 1.  
Trichrome staining

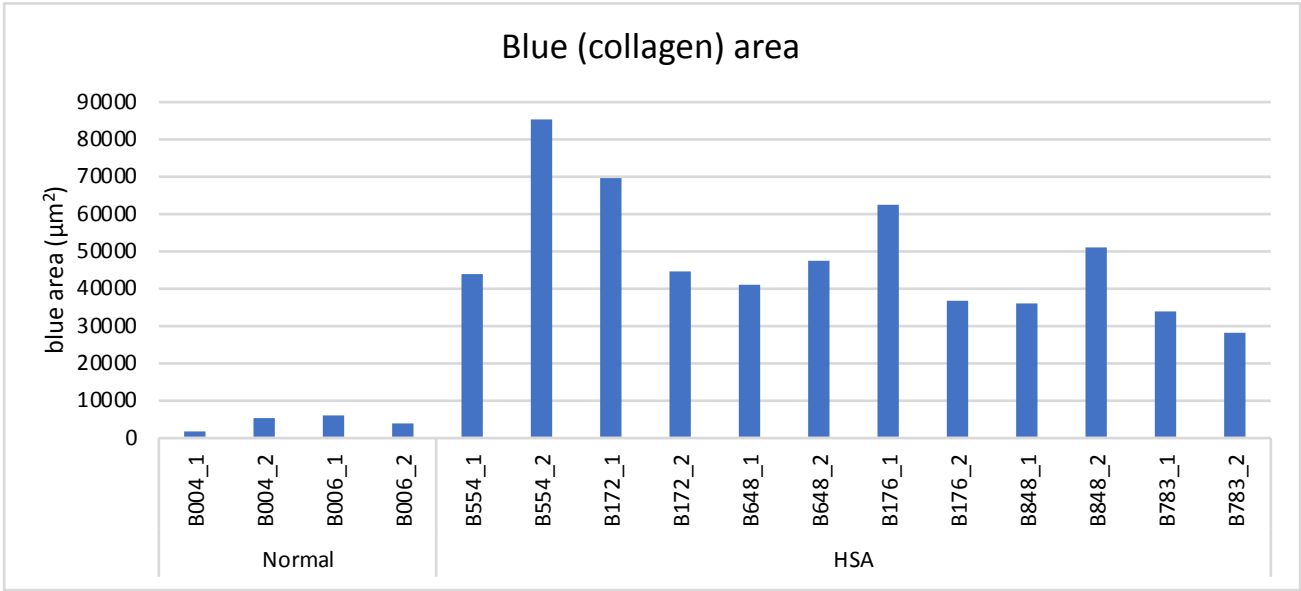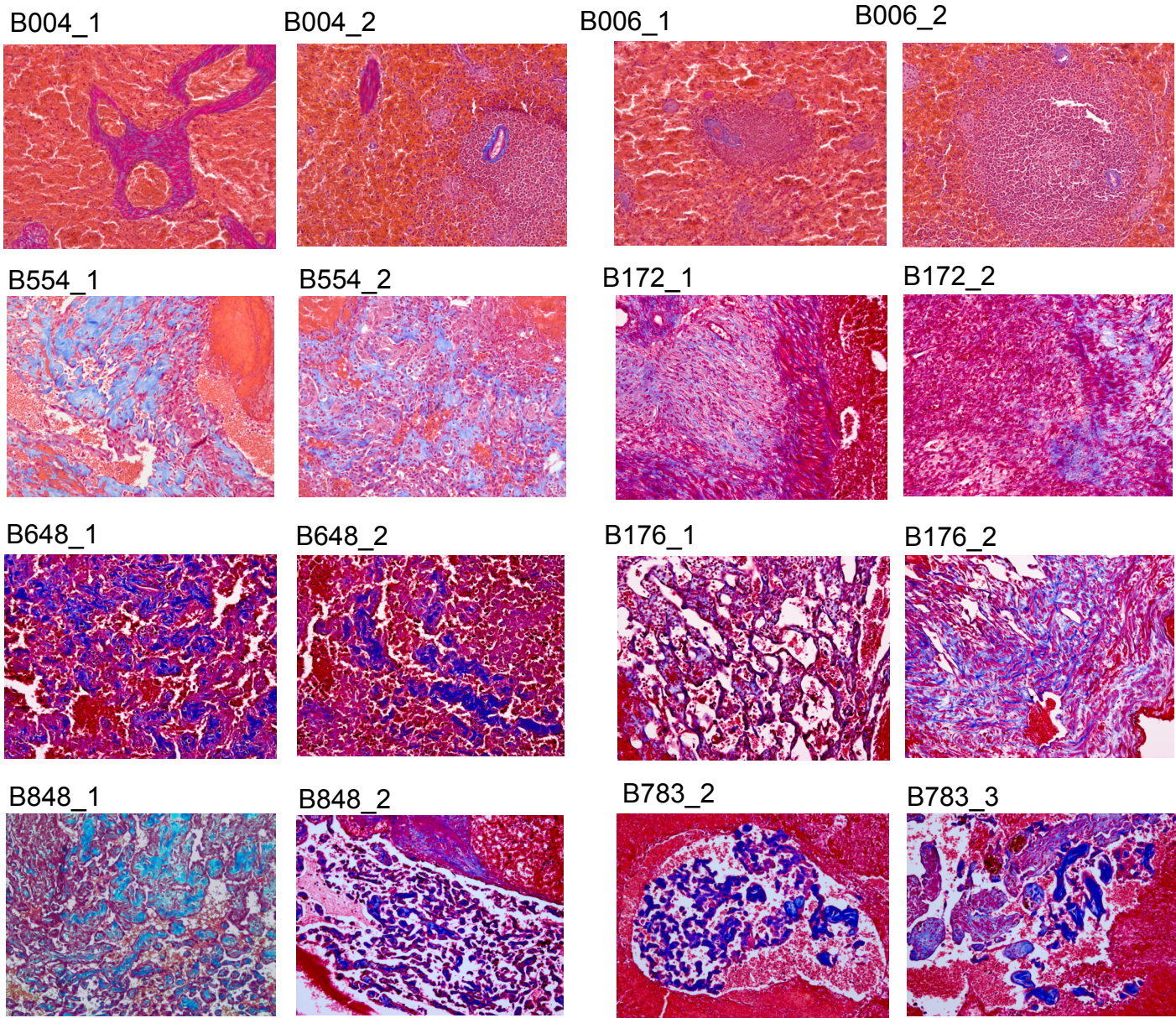

Supplement: Supplementary file 3 — Additional file 3. FS1. Trichrome staining quantification. [file 12917_2020_2395_MOESM3_ESM.pdf]

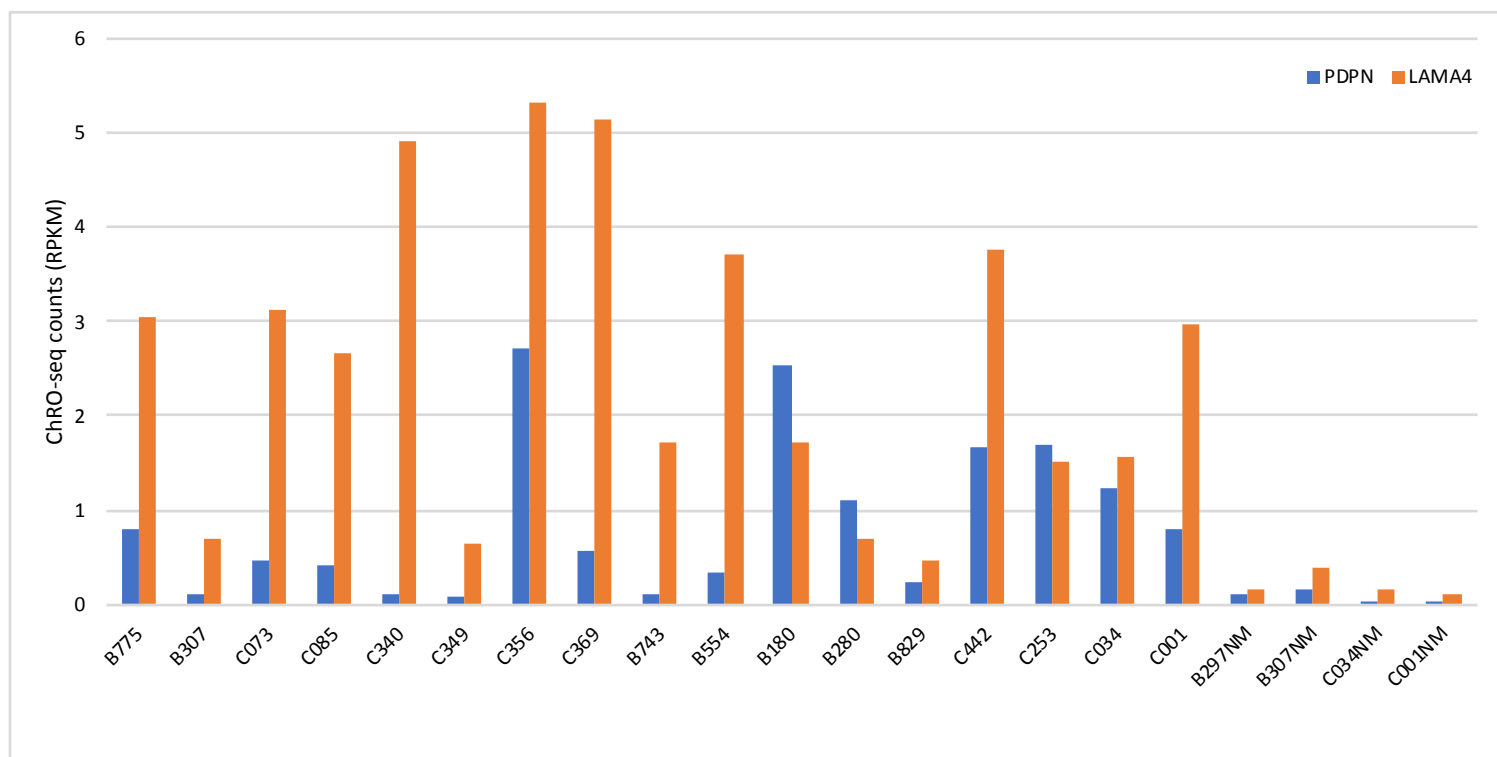

Supplemental figure 2: ChRO-seq counts on PDPN and LAMA4 gene bodies.

Supplement: Supplementary file 4 — Additional file 4. FS2. ChRO-seq counts on PDPN and LAMA4 gene bodies. [file 12917_2020_2395_MOESM4_ESM.pdf]

Supplemental Figure 3A.  
PDPN IHC

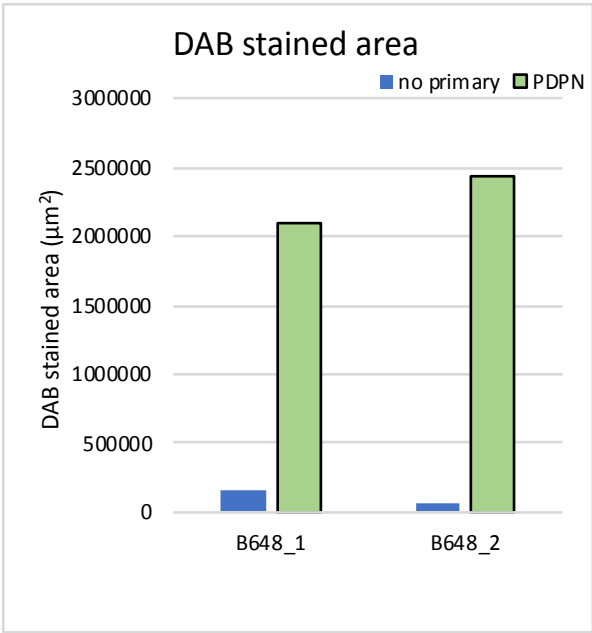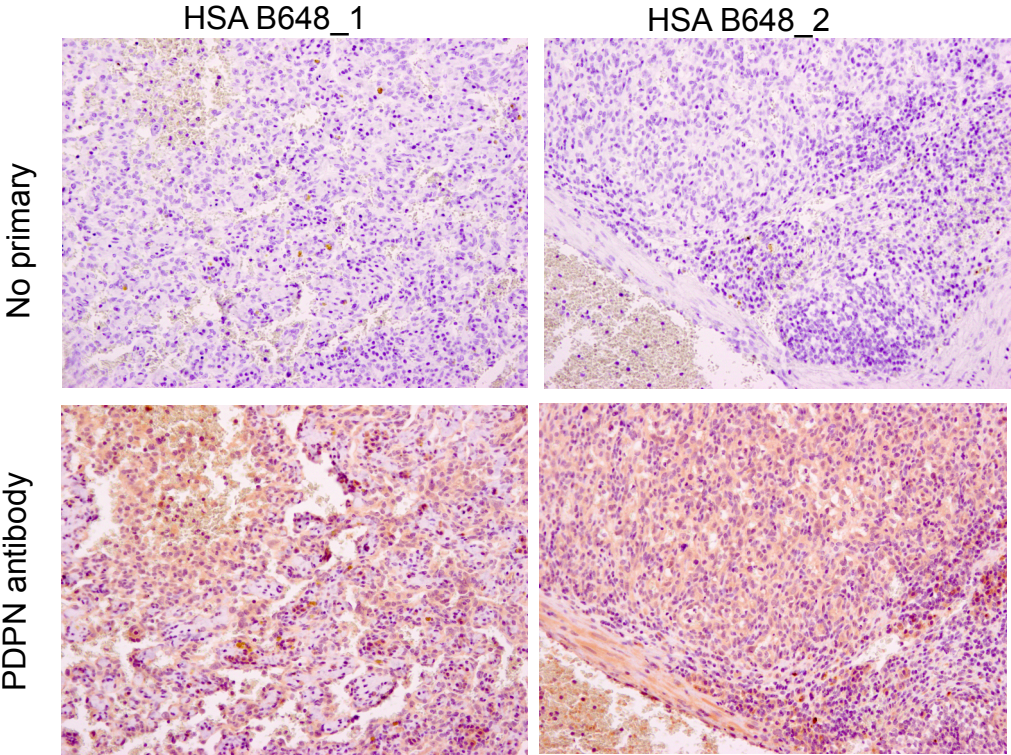

Supplement: Supplementary file 5 — Additional file 5. FS3. PDPN immunohistochemistry quantification. [file 12917_2020_2395_MOESM5_ESM.zip › SF3A_PDPN_1.pdf]

Supplemental Figure 3B.  
PDPN IHC

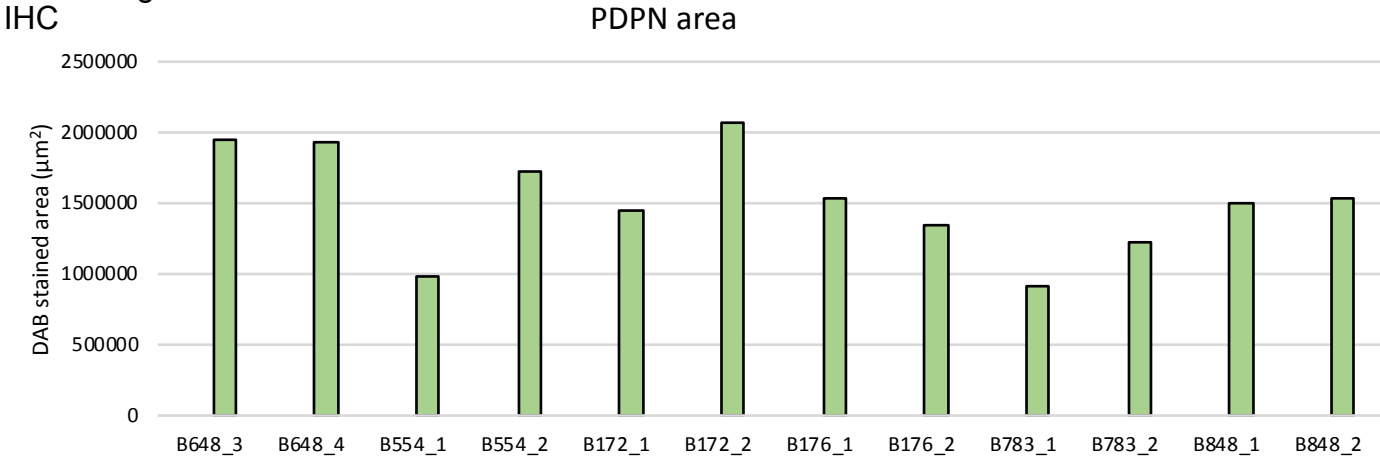

B648\_1

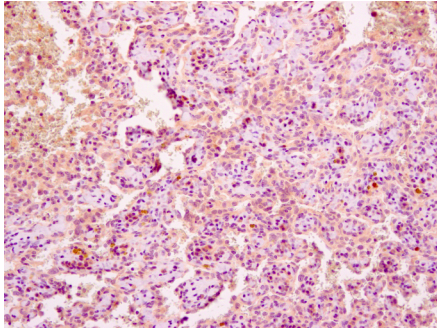

B554\_1

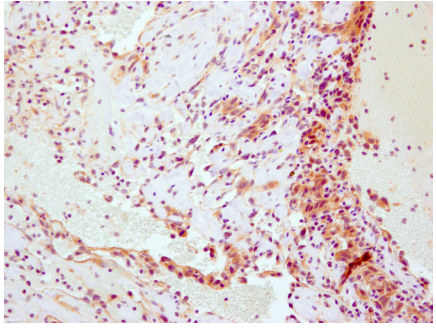

B172\_1

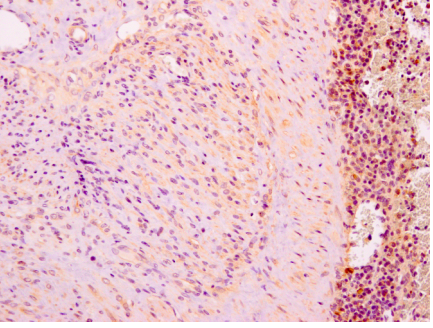

B648\_2

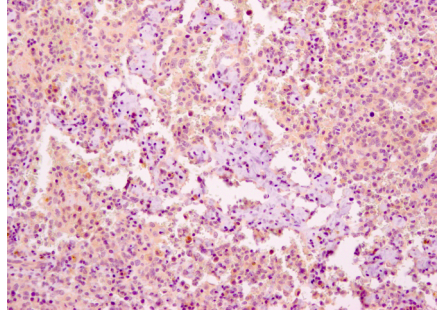

B554\_2

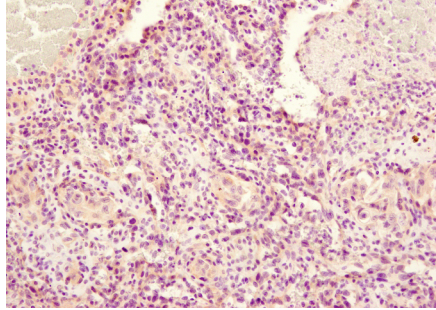

B172\_2

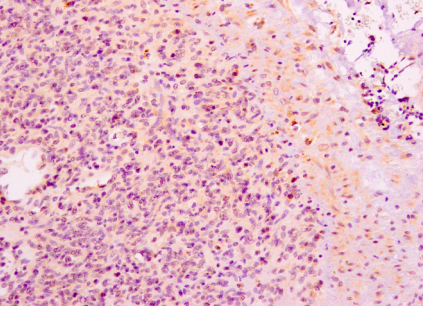

B176\_1

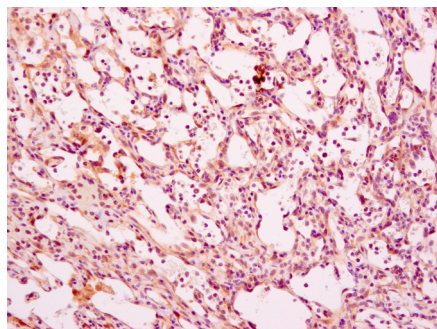

B783\_1

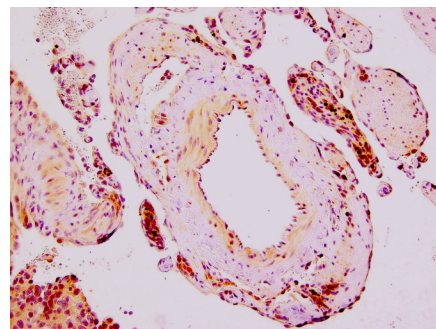

B848\_1

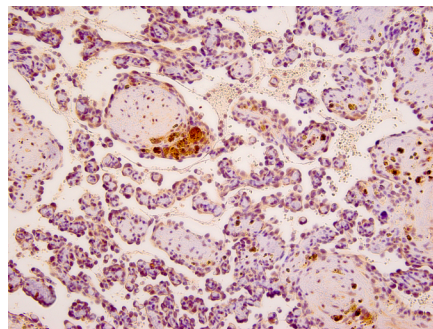

B176\_2

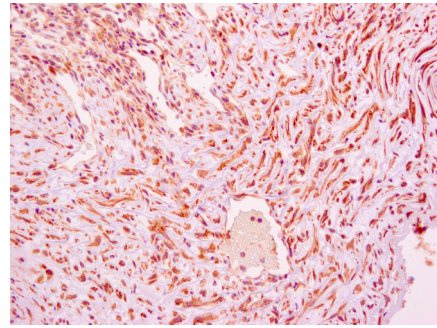

B783\_2

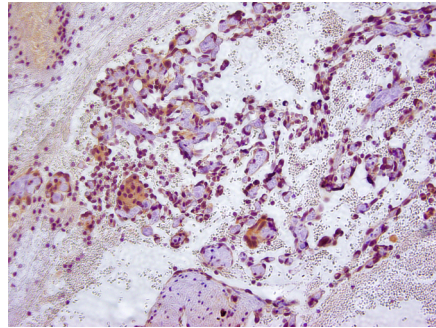

B848\_2

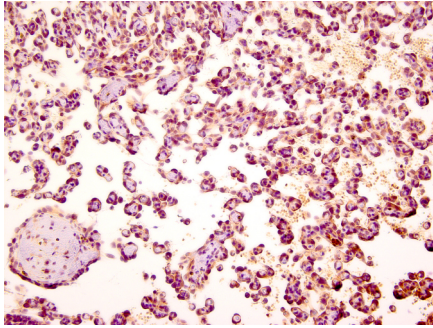

Supplement: Supplementary file 5 — Additional file 5. FS3. PDPN immunohistochemistry quantification. [file 12917_2020_2395_MOESM5_ESM.zip › SF3B_PDPN_2.pdf]

Supplemental Figure 4A. LAMA4 IHC

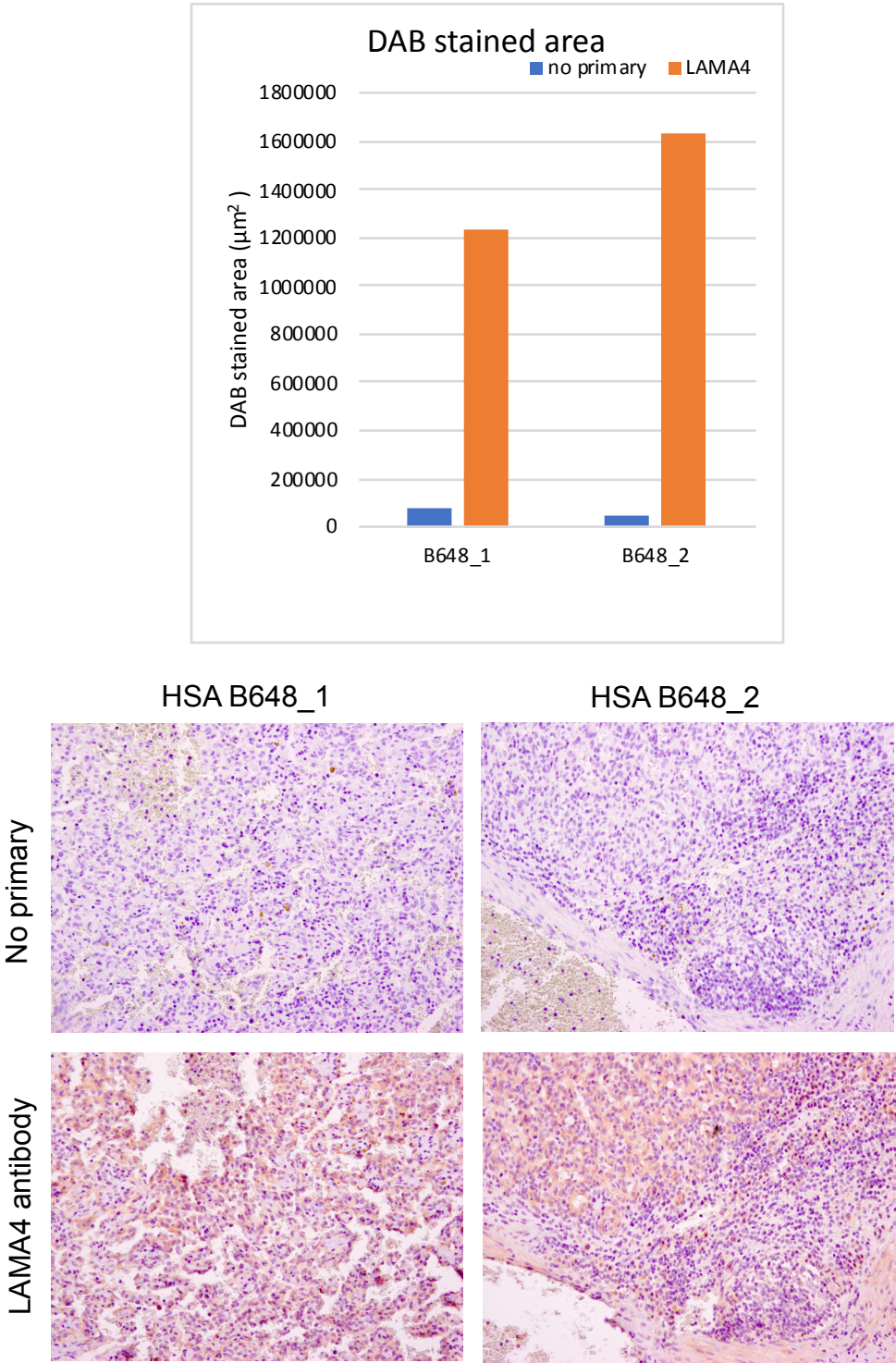

Supplement: Supplementary file 6 — Additional file 6. FS4. LAMA4 immunohistochemistry quantification. [file 12917_2020_2395_MOESM6_ESM.zip › SF4A_LAMA4_1.pdf]

Supplemental Figure4B.LAMA4 IHC

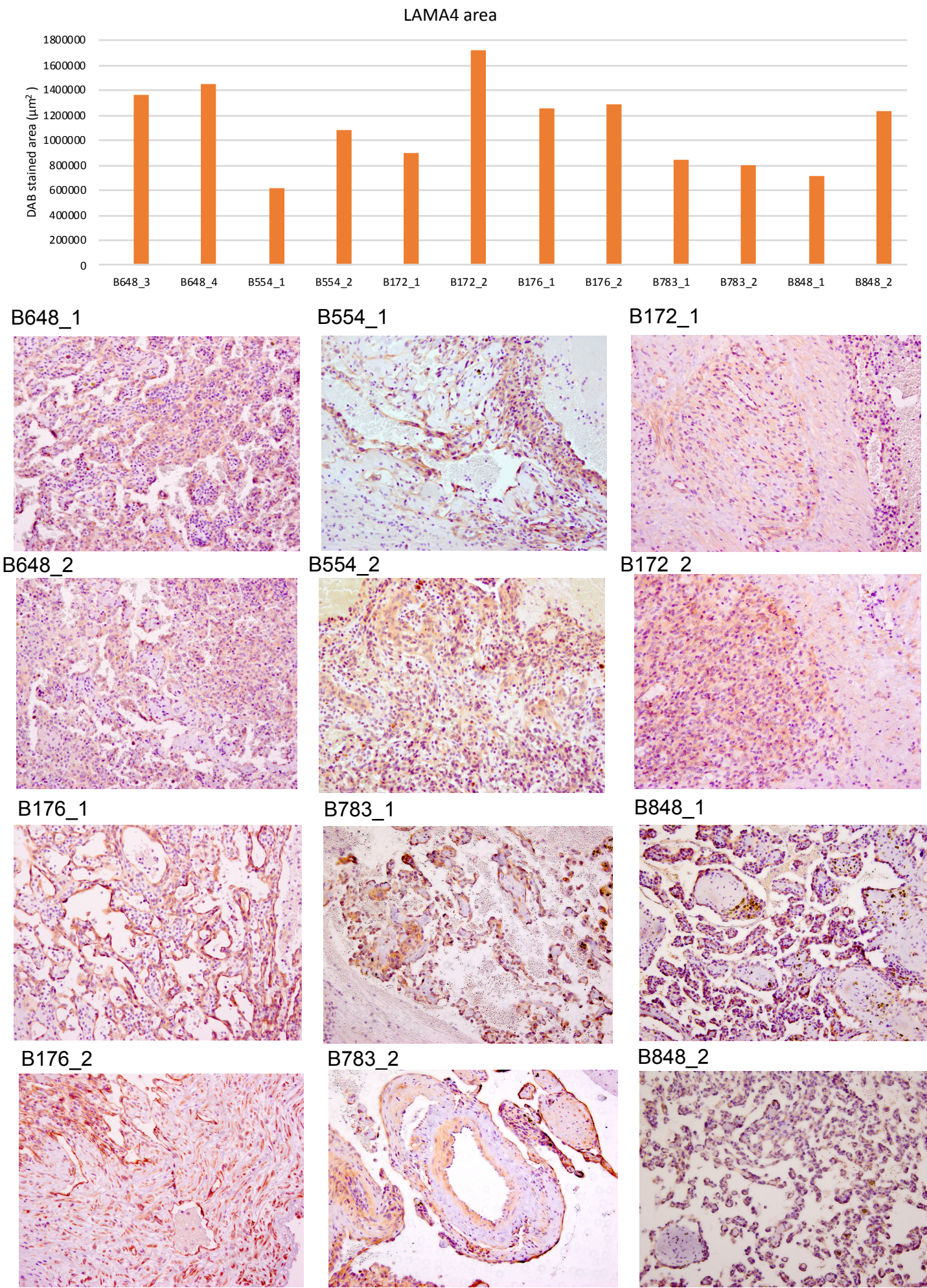

Supplement: Supplementary file 6 — Additional file 6. FS4. LAMA4 immunohistochemistry quantification. [file 12917_2020_2395_MOESM6_ESM.zip › SF4BLAMA4_2.pdf]
