# Supplementary material for: Chromatin run-on sequencing analysis finds that ECM remodeling plays an important role in canine hemangiosarcoma pathogenesis
Source: BMC Vet Res. 2020 Jun 22;16:206. doi: 10.1186/s12917-020-02395-3 (PMC7310061; doi:10.1186/s12917-020-02395-3)
Supplement: Supplementary file 8 — Additional file 8. FS6. Masson’s trichrome staining and IHC on serial sections from case B554 (HSA). [file 12917_2020_2395_MOESM8_ESM.pdf]

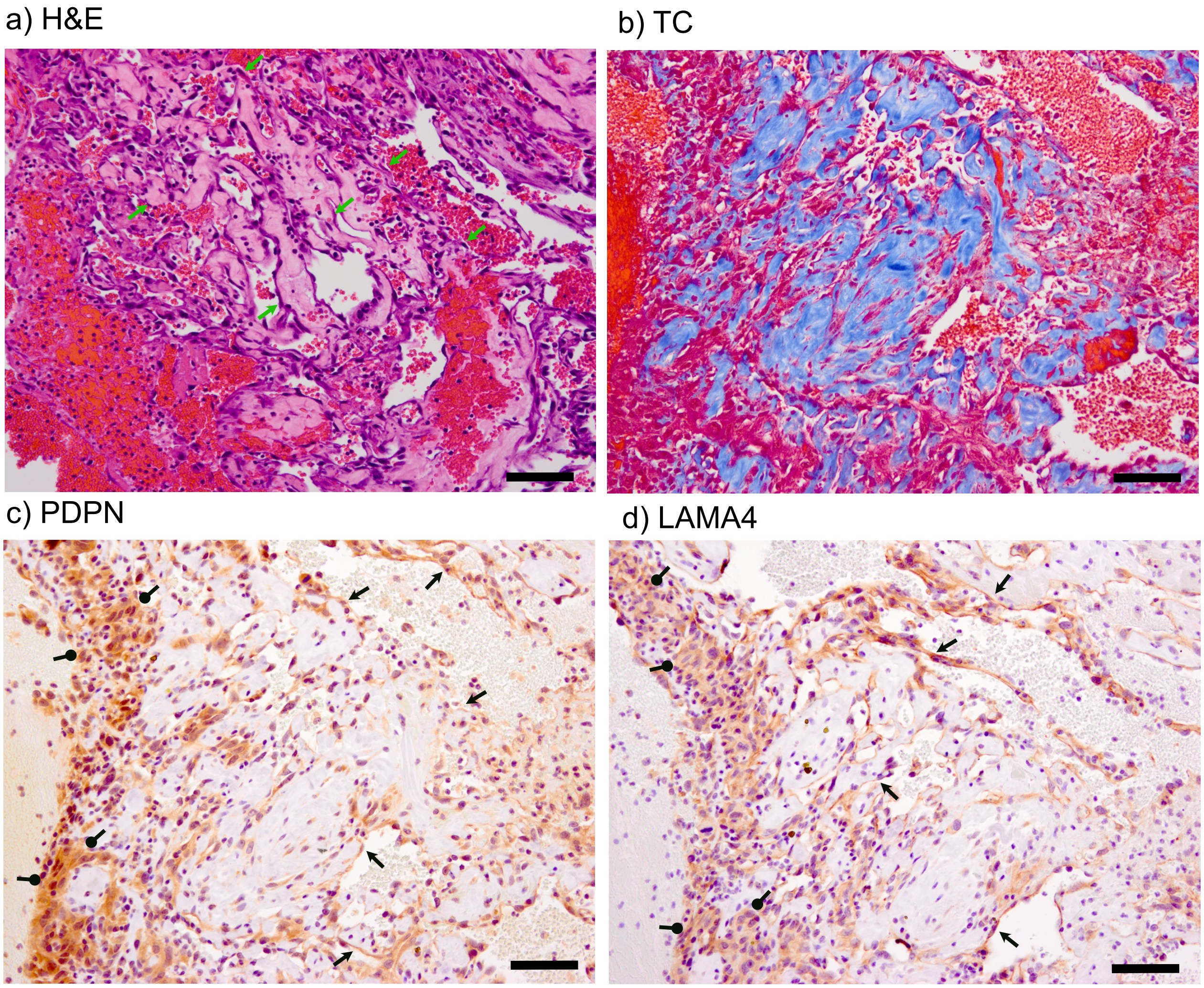

Figure S6 Masson's trichrome staining and IHC of serial sections from HSA sample (B554).
