# Supplementary material for: Chromatin run-on sequencing analysis finds that ECM remodeling plays an important role in canine hemangiosarcoma pathogenesis
Source: BMC Vet Res. 2020 Jun 22;16:206. doi: 10.1186/s12917-020-02395-3 (PMC7310061; doi:10.1186/s12917-020-02395-3)
Supplement: Supplementary file 9 — Additional file 9. FS7. Original gel images for Fig. 4b. [file 12917_2020_2395_MOESM9_ESM.pdf]

Supplemental figure FS7.
Original gel pictures for Figure 4B. Lanes 1-2, and 12-15 were cropped to make Figure 4B. Lanes 4-11 were non-HSA dog tissue samples.

Figure 4B

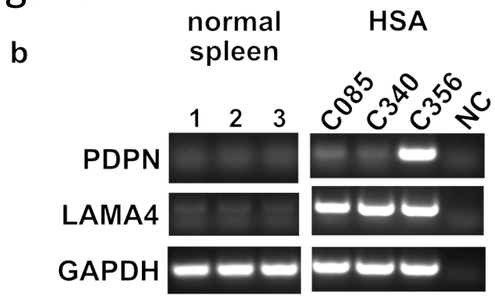

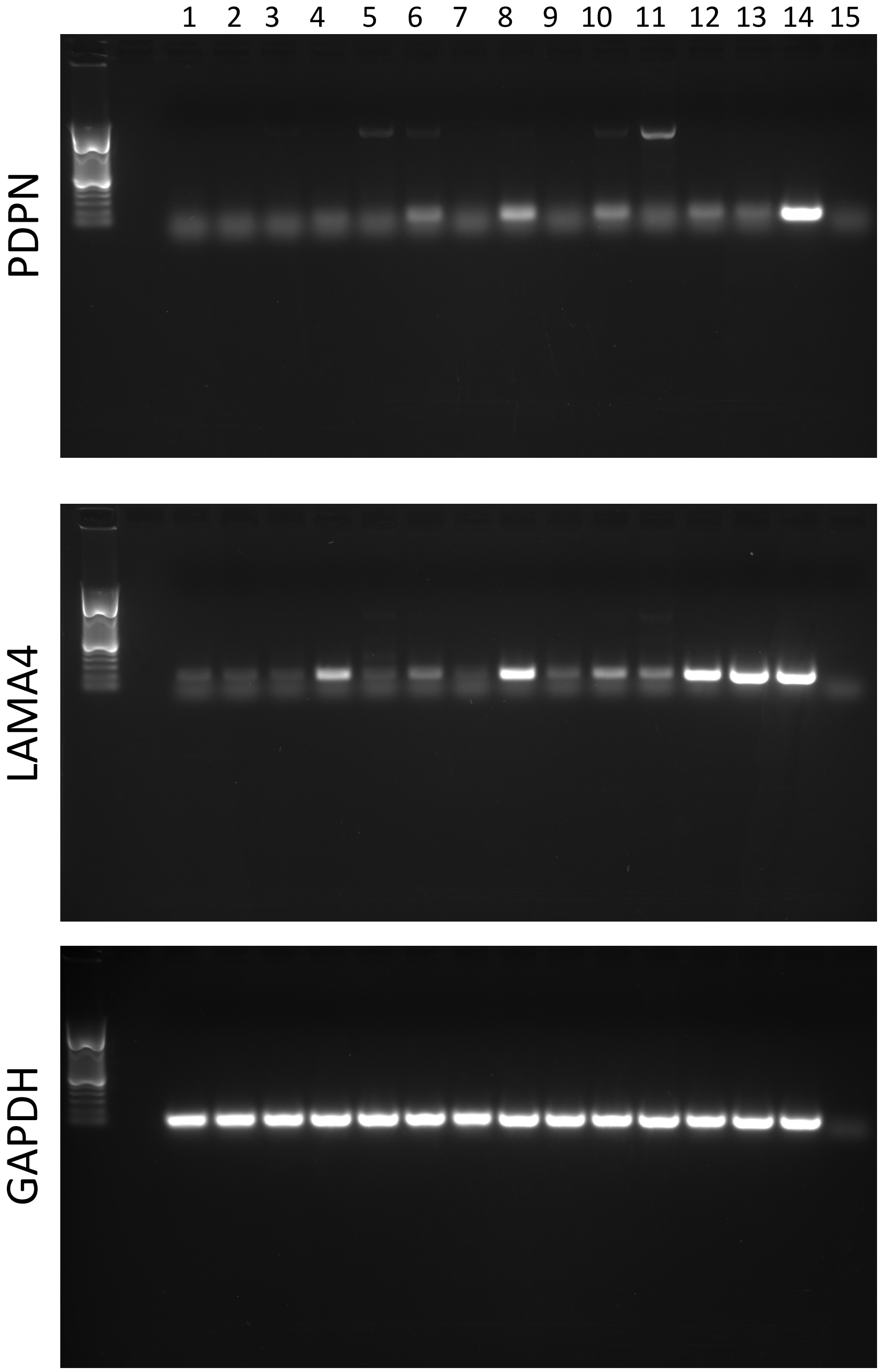

1. Normal spleen B001
2. Normal spleen B004
3. Normal spleen B006
4. Dog tissue
5. Dog tissue
6. Dog tissue
7. Dog tissue
8. Dog tissue
9. Dog tissue
10. Dog tissue
11. Dog tissue
12. HSA C085
13. HSA C340
14. HSA C356
15. No DNA
